# AlkaPlorer: A database‐driven explorer for natural alkaloids and derivatives

**DOI:** 10.1111/jipb.70173

**Published:** 2026-02-05

**Authors:** Jiahao Li, Tao Zeng, Hongquan Xu, Xu Kang, Minghui Liang, Ruibo Wu

**Affiliations:** ^1^ School of Pharmaceutical Sciences Sun Yat‐sen University Guangzhou 510006 China; ^2^ School of Pharmaceutical Sciences Hainan University Haikou 570228 China; ^3^ School of Pharmaceutical Sciences Guangzhou Medical University Guangzhou 511436 China

**Keywords:** AI‐driven applications, alkaloids, chemical space, natural products, phytochemistry

## Abstract

Alkaloids, renowned for their pivotal physiological roles in plant defense and chemical medium, constitute a structurally diverse class of bioactive natural products with substantial therapeutic potential in modern drug development. There is currently no dedicated alkaloid database, highlighting an urgent need for such a resource. Here, we present AlkaPlorer (https://alkaplorer.qmclab.com/), the first systematic alkaloid database, which has compiled over 130,000 alkaloids from 12,250 species, with reported activity against 6,583 biological targets. AlkaPlorer not only integrates comprehensive experimentally validated data and computationally predicted properties for each alkaloid, but also establishes standardized notation and associations among various data elements, forming a correlative‐type dataset. Extensive chemoinformatic analyses on structural scaffolds, biosynthetic precursors, physicochemical properties, and phylogenetic distributions across plant taxa are performed based on AlkaPlorer, providing new insights into the chemical diversity, structural evolution, and biosynthetic regularity of plant alkaloids. AlkaPlorer enables easy access and efficient retrieval and provides a foundational resource for AI‐driven applications in plant metabolism and alkaloid research.

## Graphical abstract

The alkaloid database AlkaPlorer integrates over 130,000 compounds across 12,250 species. By linking chemical structures with biological and evolutionary data, it reveals how these molecules evolve and function. This platform serves as a vital resource for AI‐driven discovery in plant metabolism and modern drug development.

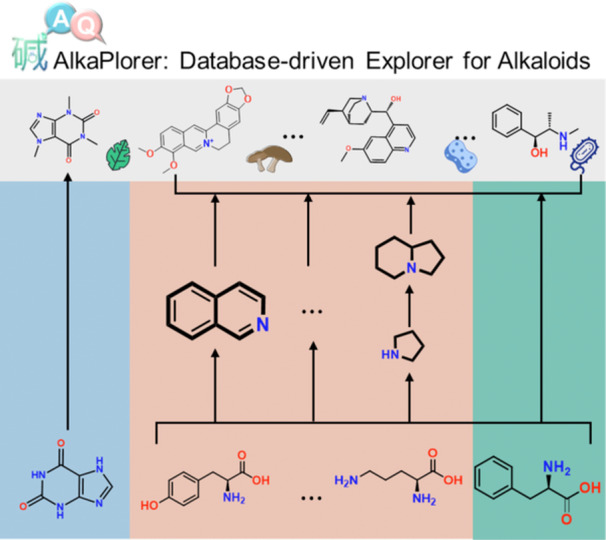

## INTRODUCTION

**Figure 1:**
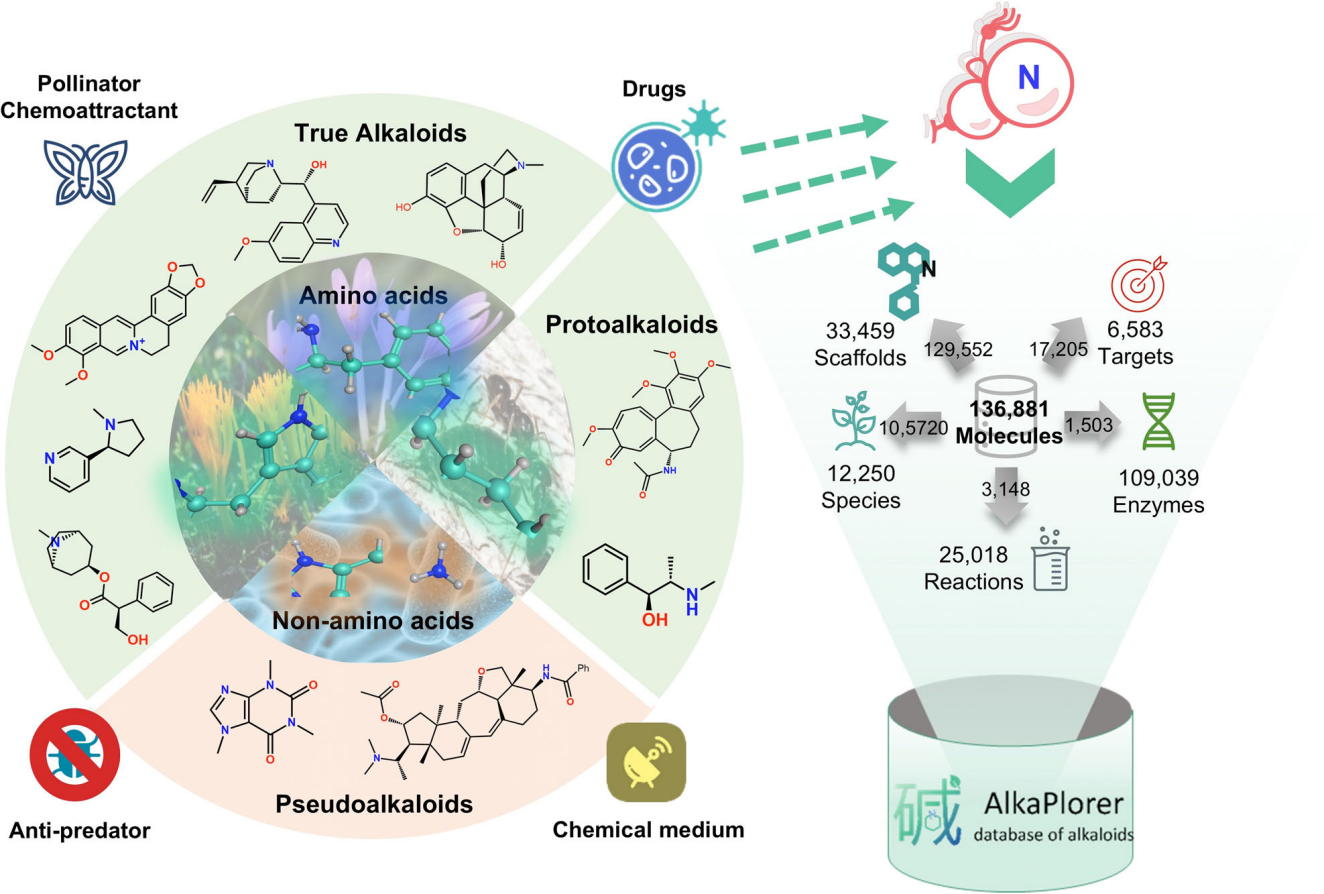
The framework and data integration workflow of the AlkaPlorer database

Alkaloids are a group of natural compounds, occurring as specialized metabolites with nitrogen as a characteristic element present in their structures (Dey et al., 2020; Bhambhani et al., 2021). The biogenesis of alkaloids primarily originates from amino acid precursors such as ornithine (Leete, 1964; Philipov and Doncheva, 2013; Funayama and Cordell, 2015) and tyrosine (Khan et al., 2013). These are known as true alkaloids (nitrogen atom in heterocyclic ring) and protoalkaloids (nitrogen in a side chain or open‐chain form). While non‐amino acid precursors such as xanthine (Ashihara et al., 2017) also contribute to their formation (known as pseudoalkaloids, Figure 1). As one of nature's most prolific secondary metabolites, alkaloids exhibit an exceptionally vast chemical space characterized by both structural specificity and unparalleled diversity. For a long time, alkaloids have attracted much attention because of their outstanding contributions in drug discovery, such as morphine (Inturrisi, 2002; Devereaux et al., 2018; Wicks et al., 2021), a classic analgesic agent extracted from *Papaver somniferum*, and camptothecin (Kamle et al., 2024), which is used to treat various cancer types. While the biological significance of alkaloids goes far beyond this, they also perform rich and complex ecological functions in nature, such as defending against predation, inhibiting pathogens, regulating signal transduction, mediating symbiosis, etc., demonstrating significant adaptive evolutionary characteristics (Cushnie et al., 2014; Marques da Fonseca et al., 2023). These functions are closely related to their skeleton structure. For example, pyrrole and pyridine alkaloids are often involved in the process of neural activity. Indoles are widely found in plant disease and insect defense (Sun et al., 2024), and quinolines are representative in microbial antagonism (Kumar et al., 2023). These skeletal differences not only reflect the phylogenetic trajectory of alkaloids but also provide a basis for a deeper understanding of their ecological functions and evolutionary pathways (Wink, 2003).

In recent years, with the rapid development of separation and purification techniques, spectral analysis, and bioinformatics, research on alkaloids has continued to deepen, not only revealing more structural types with important pharmacological activities but also gradually discovering their increasingly diverse functions in ecosystems. For instance, the structural diversity of isoquinoline alkaloids continues to expand, while their biological activity and synthesis strategies are also constantly optimized (Yang et al., 2024). Gonzalez et al. first characterized toxic alkaloids from cryptic dendrobatid *Silverstoneia punctiventris*, revealing not only their potential role in gustatory defense upon contact but also their possible function as volatile compounds detectable at a distance (Gonzalez et al., 2021). From the perspective of systematics and evolutionary ecology, the diversity of alkaloid skeletons reflects their adaptive evolutionary pathways in different species. Skeletons such as indole, isoquinoline, piperidine, and pyrrolidine exhibit enrichment trends in specific families and genera, showing species preference. This type of information is of great value for understanding the biosynthesis evolution and species functional differentiation of natural products (NPs).

With the development of cheminformatics and high‐throughput NPs mining technology, multiple NP databases have emerged (Sorokina and Steinbeck, 2020; Zeng et al., 2024). Subscription‐based Dictionary of Natural Products (DNP) (Harborne and Hall, 1994) contains more than 360,000 entries. Natural Products Atlas (van Santen et al., 2022; Zhao et al., 2023) catalogs 32,552 bacterial and fungal metabolites, while NPASS integrates about 100,000 entries with activity and species‐source annotations. COCONUT (Chandrasekhar et al., 2025) is a collection of NP databases featuring over 400,000 unique compounds, in which 167,000 can be classified as alkaloids by NPClassifier (Kim et al., 2021). These databases integrate a large amount of information on the structure, activity, and sources of NPs, providing important support for the discovery and redevelopment of natural medicines, but lack dedicated taxonomic characterization of alkaloids. Although there are some alkaloid‐specific databases, such as BIAdb (a database for benzylisoquinoline alkaloids) (Singla et al., 2010) and Peptaloid (a database for exploring peptide alkaloids) (Behera et al., 2024), only a part of the alkaloids are covered. In the meantime, current systems employ heterogeneous classification criteria; for example, some classifications rely on phylogenetic origin (e.g., Amaryllidaceae alkaloids (Cimmino et al., 2017), Solanum alkaloids (Heretsch and Giannis, 2015)), while others emphasize structural motifs (e.g., indole alkaloids (Rosales et al., 2020)). This inconsistency has led to overlapping classifications and compromised the taxonomic significance of alkaloid categorization in existing databases. Notably, although the biosynthetic‐structural taxonomy provides a comprehensive framework integrating biosynthetic origins with molecular architecture, this approach has yet to be adopted by existing databases (Seneca, 2007; Bhambhani et al., 2021). Furthermore, current databases exhibit fragmented data dimensions, compartmentalizing structural biosynthesis, bioactivity profiles, and enzymatic pathway annotations without systematic integration, making it difficult to support in‐depth chemical space exploration, skeleton evolution analysis, and species association research.

To address these limitations, a specialized database for alkaloids and their derivatives has been established, and a broad spectrum of annotations, such as skeleton classification, biological source, metabolism, and activities, has been integrated. We identified lineage‐specific enrichment patterns by performing large‐scale scaffold and biosynthetic origin analysis, and explored the correlations between alkaloid structures and their producing species. In addition, comprehensive cheminformatic profiling was conducted to assess drug‐likeness and bioactivities across structural categories. These analyses not only facilitate a more standardized classification system but also enable in‐depth exploration of chemical evolution, species‐specific biosynthesis, and ecological functions. A user‐friendly web server (AlkaPlorer) has also been developed, where the database can be freely accessed, and the chemical space of alkaloids can be systematically explored by researchers in this field. The comprehensive, dedicated, interconnected AlkaPlorer can be a promising tool for alkaloid discovery and biosynthesis.

## RESULTS

### Data contents

AlkaPlorer contains 136,881 non‐redundant alkaloid molecules rigorously curated from literature and public databases, encompassing 33,459 unique scaffolds. Six thousand five hundred and eighty three pharmacological targets, 25,018 metabolic reactions, and 109,039 biosynthetic enzymes were integrated to bridge chemical structures with biological pathways (Figure 1). Seven thousand three hundred and eighty alkaloids in AlkaPlorer are collected manually, while the remaining data, integrated from public databases, were not manually curated.

**Figure 2:**
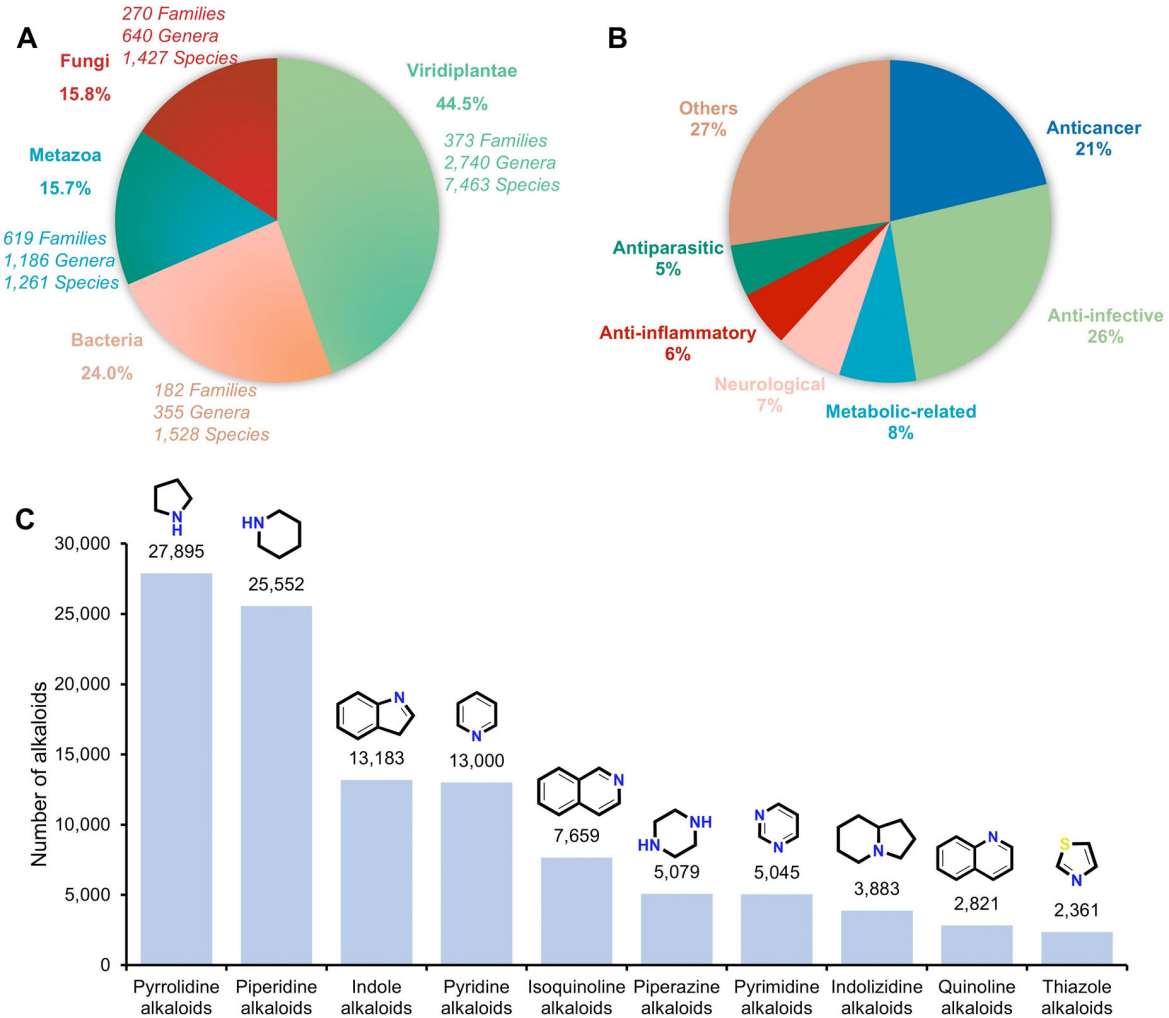
Comprehensive overview of the AlkaPlorer database **(A)** Taxonomic breakdown of the biological sources from which alkaloids were compiled, with the number of families, genera, and species with definitive taxonomic assignments per kingdom. **(B)** Therapeutic Potential Classification by Clinical Indication. **(C)** List of top 10 nitrogen‐containing heterocyclic scaffolds in alkaloids and their quantities. A representative chemical structure is shown above each corresponding bar.

Taxonomic profiling of the alkaloid sources (Figure 2A) reveals 11,679 species with definitive taxonomic assignments, spanning Viridiplantae (44.5%), Bacteria (24.0%), Metazoa (15.7%), and Fungi (15.8%), demonstrating their phylogenetic diversity (Figure 2A). We summarized the top 10 alkaloid‐producing families within each taxonomic group (Viridiplantae, Fungi, Bacteria, Metazoa) to characterize the overall biosynthetic source distribution of alkaloids (Figure S1). Among plant taxa, the Apocynaceae, which is well known as an alkaloid‐rich family (Bhadane et al., 2018), demonstrates the highest alkaloid abundance, followed by Fabaceae, Asteraceae, Ranunculaceae, and Rutaceae. The Aspergillaceae and Streptomycetaceae family exhibit the dominant abundance in fungal and bacterial alkaloids, respectively, indicating a notable clustering of biosynthetic capacity. In Metazoa, human‐associated alkaloids represent the most prevalent subgroup, whereas marine sponges exhibit the broadest taxonomic distribution of these compounds. Seventeen thousand two hundred and five compounds have been reported with various biological activities, including anticancer, anti‐infective, metabolic‐related, neurological, and so on (Figure 2B), most of which act on organisms, with the rest acting on single proteins or cell lines (Table S1). Anti‐infective and anticancer activities collectively represent nearly half of all reported bioactivities, establishing them as the primary pharmacological profiles of alkaloids. Since the compounds in AlkaPlorer were classified by biosynthetic‐structural taxonomy, the top 10 alkaloid classes are shown in Figure 2C. Pyrrolidine and piperidine alkaloids each exceed 25,000 characterized compounds, which provides strong evidence that five‐ and six‐membered saturated nitrogen heterocycles constitute the predominant structural motifs for nitrogen incorporation in alkaloid architectures. Indole alkaloids, biosynthesized from tryptophan or tryptamine precursors, derive their structural uniqueness from the indole nucleus—a privileged pharmacophore in medicinal chemistry (Sravanthi and Manju, 2016). Pyridine alkaloids, containing the pyridine scaffold, have been extensively utilized in developing clinical candidates due to their capacity for multi‐center substitution. This structural feature not only enhances pharmacological potency but also optimizes pharmacokinetic properties (De et al., 2022). It is worth noting that these high‐frequency structures frequently appear in top nitrogenous scaffolds of U.S. FDA‐approved drugs, such as piperidine (72 drugs, 1st), pyridine (62 drugs, 2nd), piperazine (59 drugs, 3rd), pyrrolidine (37 drugs, 5th), thiazole (30 drugs, 6th), indole (17 drugs, 9th), and pyrimidine (16 drugs, 10th), revealing the structural relevance of alkaloid‐inspired designs in pharmaceutical development (Vitaku et al., 2014). Notably, isoquinoline alkaloids (7,659 molecules), which rank fifth in natural abundance, exhibit broad pharmacological activities—including antitumor, anti‐infective, and anti‐inflammatory effects—that highlight their underutilized therapeutic potential (Shang et al., 2020; Wang et al., 2024). This significance is further corroborated by the inclusion of tetrahydroioquinoline derivatives among the top scaffolds. These statistical findings highlight the untapped potential of alkaloid‐derived architectures in contemporary drug discovery (Hong et al., 2020).

### Structure characteristics

**Figure 3:**
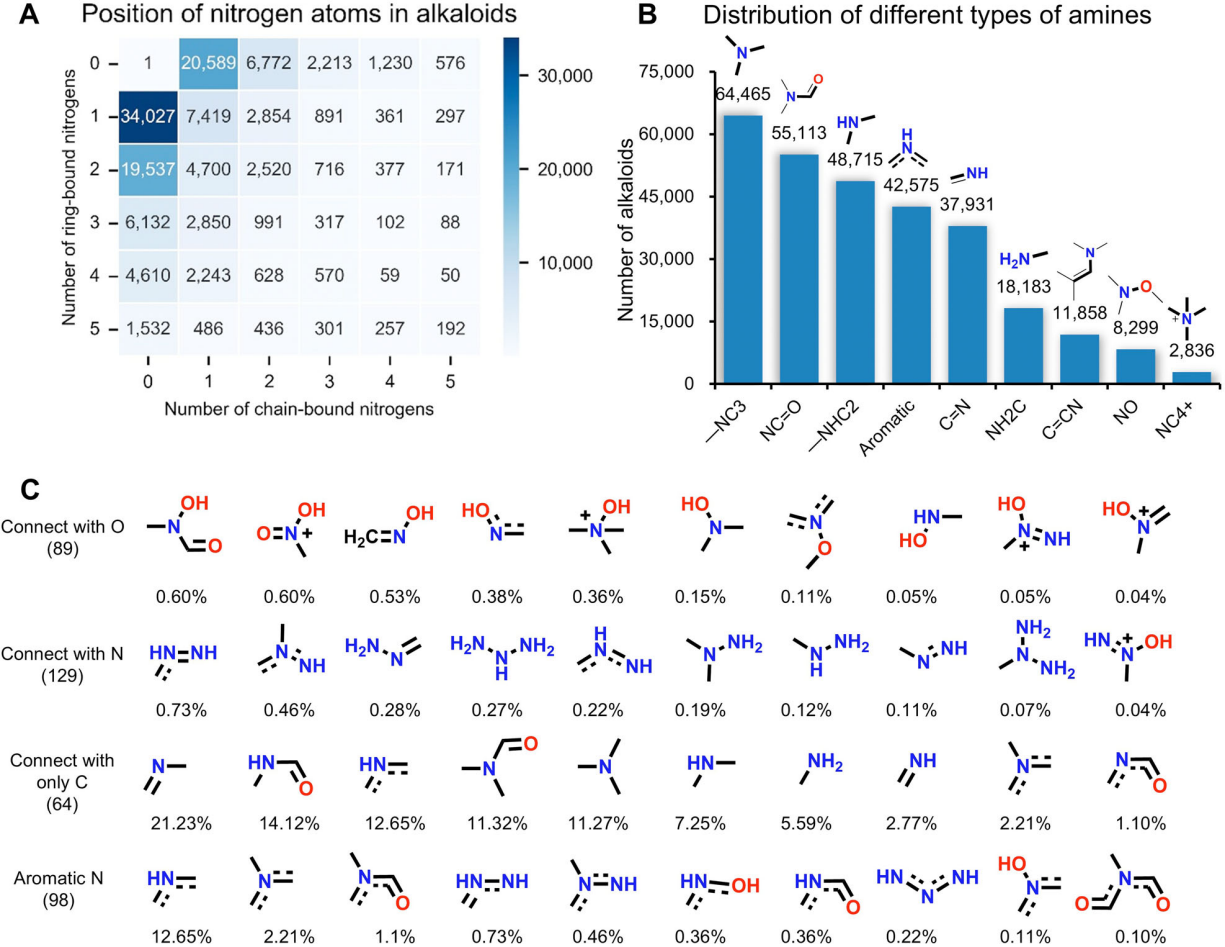
Structure and classification statistics of alkaloids **(A)** Number of nitrogen atoms and the type of bonds they form in alkaloid molecules. Each cell represents the number of alkaloids with the corresponding number of nitrogen atoms in the chain (*x*‐axis) and ring (*y*‐axis) positions. The color scale indicates the number of molecules, with darker blue representing higher numbers. **(B)** Number of alkaloid molecules harboring nine common nitrogen‐containing groups. The structure of the group is shown above each bar. **(C)** Top 10 nitrogen‐containing chemical environments per bond configuration. The number of alkaloids with each bond configuration is indicated in parentheses; the percentages below each group indicate nitrogen‐containing structure prevalence.

Figure 3A systematically displays nitrogen atom distribution patterns in alkaloid architectures through a matrix visualization and demonstrates two predominant nitrogen localization patterns in alkaloid evolution—heterocyclic nitrogen integration versus free amine group retention. The largest group of alkaloids (34,028) has a single nitrogen atom in ring structures, followed by 20,590 compounds that have one in chain configurations. This distribution profile corroborates the evolutionary preference for mono‐nitrogenated scaffolds, particularly the privileged five‐ and six‐membered ring systems previously documented. A progressive exponential decrease in alkaloid frequency is observed with increasing nitrogen atom counts, demonstrating a biochemical propensity toward low‐nitrogen‐content molecular architectures.Figure 3B delineates the heterogeneous chemical microenvironments of nitrogen atoms commonly occurring in alkaloid architectures. The tertiary amine and secondary amine structures ranked first (64,665) and third (48,715), respectively, while conjugated structures (in aromatic rings) ranked fourth (42,575). This indicates that in alkaloids, nitrogen is more likely to appear in non‐conjugated structures than conjugated ones. Furthermore, amide groups accounted for the second position (55,113), indicating that peptide alkaloids possess a unique combination of structural diversity. The amino group ranked sixth, indicating that a portion of nitrogen in alkaloids exists as side chains or at the terminal ends of molecular chains. To better investigate the chemical environments of N, we summarized all connection modes of N and categorized them into connected to oxygen (O), connected to N, connected only to carbon (C), and existing in aromatic structures (Figure 3C). The analysis revealed that the connection mode of bonding solely with C occurred most frequently, followed by N in aromatic structures, while connections with N and O showed relatively fewer instances despite their diverse modes. Specifically, the presence of a carbon atom bonded to nitrogen (C─N) that is additionally double‐bonded to oxygen (C═O) represents a unique chemical context. In the frequency distribution of amide bonds, nitrogen atoms are seldom incorporated within aromatic ring systems. Notably, amide configurations where nitrogen functions as a secondary amine dominate, accounting for 14.12% of observed cases, followed by tertiary amines at 11.32%.

**Figure 4:**
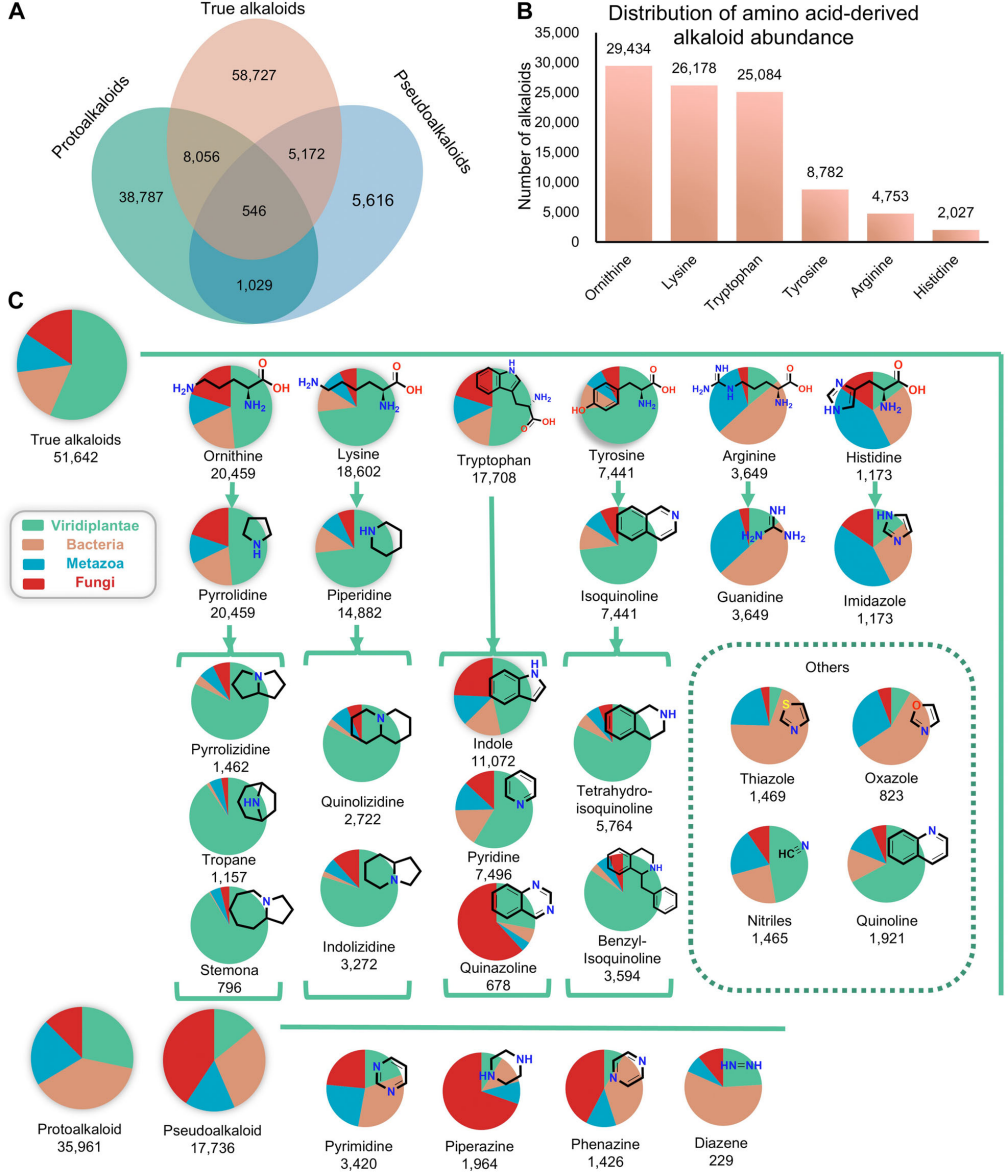
Biogenesis, structural classifications, and biological sources of alkaloids **(A)** Venn diagram showing the numbers of alkaloid types across three categories: true alkaloids, protoalkaloids, and pseudoalkaloids. **(B)** Number of alkaloid compounds derived from the different amino acid precursors ornithine, lysine, tryptophan, tyrosine, arginine, and histidine. **(C)** Biogenesis structure tree of true alkaloids, protoalkaloids, and pseudoalkaloids. Their relative distribution among the biological sources (Viridiplantae, Metazoa, fungi, bacteria) is illustrated by the pie charts.

Figure 4A quantitatively maps the interclass distribution patterns of three principal alkaloid categories. True alkaloids are the most abundant, followed by protoalkaloids, with pseudoalkaloids being the least common based on statistical distribution. This phylogenetic interconnectivity among alkaloid classes underscores the biosynthetic promiscuity across divergent metabolic pathways, particularly evident in hybrid architectures that bridge multiple biosynthetic origins. Especially, in true alkaloids, three amino acid precursors (ornithine, lysine, and tryptophan) dominate alkaloid biosynthesis at equivalent orders of magnitude (Figure 4B). This is consistent with the abundance of pyrrolidine, piperidine, indole, and pyridine‐type alkaloids derived from these precursors. As shown in Figure 4C, true alkaloids predominantly originate from plant‐derived biosynthetic pathways, constituting over 50% of their biogenetic sources. In contrast, protoalkaloids and pseudoalkaloids exhibit higher representation in microbial‐derived alkaloids. Pyrrolidine alkaloids, biosynthesized from L‐ornithine, represent the most abundant class of alkaloids in AlkaPlorer. These compounds exhibit broad phylogenetic distribution across Viridiplantae, Metazoa, Fungi, and Bacteria. Notably, the characteristic scaffolds—including tropane, pyrrolizidine, and stemona alkaloids—show preferential occurrence in plant lineages. Exemplified by pyrrolizidine alkaloids, these specialized metabolites demonstrate significant enrichment in taxonomically diverse angiosperm families such as Asteraceae, Boraginaceae, and Fabaceae (Tamariz et al., 2018). Piperidine alkaloids (biosynthesized from L‐lysine) and isoquinoline alkaloids (derived from L‐tyrosine) exhibit significantly broader phylogenetic distribution patterns within Viridiplantae lineages compared to other taxonomic groups. Phylogenetic analyses position isoquinoline alkaloids as evolutionarily conserved specialized metabolites in ancient vascular plants (Liscombe et al., 2005). Their biosynthesis shows primary localization within basal angiosperms of the order Ranunculales, particularly in the families Ranunculaceae, Berberidaceae, Papaveraceae, and Fumariaceae (Singh et al., 2021). Guanidine alkaloids biosynthesized from L‐arginine dominate bacterial alkaloid profiles, accounting for over 50% of their taxonomic distribution (Berlinck, 2002). Imidazole, derived from histidine, frequently appears in animal‐derived sources, primarily attributable to their prevalence in sponges (Porifera) and other marine invertebrates (e.g., Cnidaria, Mollusca), which produce diverse imidazole‐containing compounds (Jin, 2016). Alkaloid scaffolds with unclear biosynthetic origins, such as thiazole alkaloids and oxazole alkaloids, primarily originate from bacterial sources and marine invertebrates, including sponges and ascidians (Jin, 2016).

### Drug‐like properties and activities

**Figure 5:**
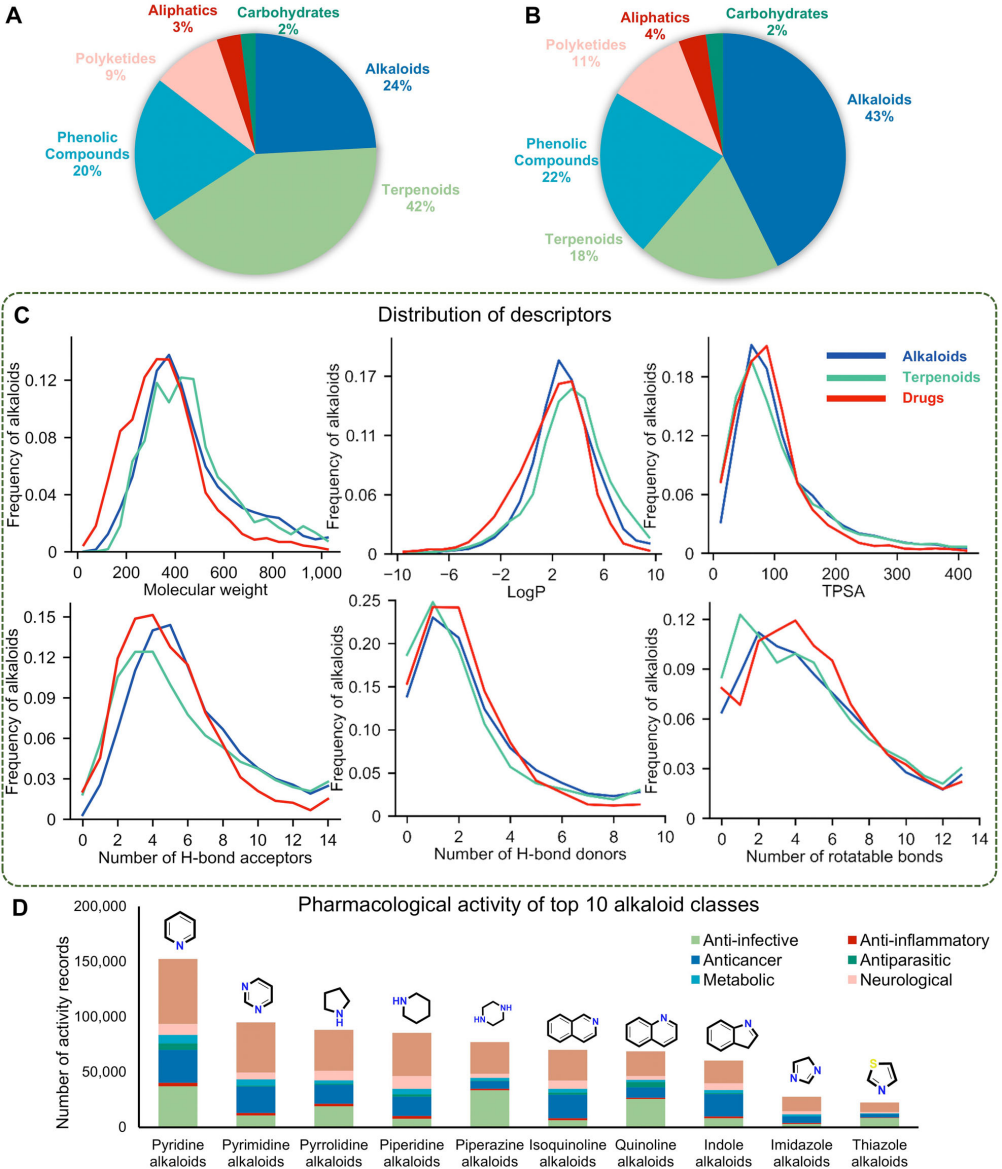
Comprehensive comparative analysis of alkaloids with other natural products and approved drugs, and the bioactivity distribution across major alkaloid scaffolds **(A)** Types of natural products (NPs) cataloged in the Dictionary of Natural Products (DNP). **(B)** Types of NP‐derived small‐molecule drugs in DrugBank (329 molecules, version 5.1.10), sorted by structure. **(C)** Comparative analysis of physicochemical parameters (molecular weight [MW], lipophilicity [LogP], topological polar surface area [TPSA], hydrogen‐bond acceptor [HBA], hydrogen‐bond donor [HBD], and rotatable bonds) among alkaloids (AlkaPlorer), terpenoids (TeroKit), and approved drugs (DrugBank, version 5.1.12). **(D)** Bioactivity of the 10 most prevalent alkaloid classes. The different pharmacological uses are shown in different colors.

As shown in Figure 5A, terpenoids and alkaloids are two of the largest classes of natural products, while intriguingly, although alkaloids represent numerically smaller groups compared to terpenoids in NP inventories, they account for a larger proportion in drugs (Figure 5B). This disparity highlights the critical role of alkaloid‐derived scaffolds in drug development, suggesting inherent advantages in their pharmacokinetic properties or target specificity compared to terpenoid‐based compounds.

To investigate the reasons behind the superior drug‐likeness of alkaloids compared to terpenoids, Figure 5C shows the distribution of physicochemical properties of interest in AlkaPlorer, including the well‐known “rule of five” (RO5) reported by Lipinski et al., which is a set of guidelines that define physicochemical conditions of orally active drugs (Lipinski et al., 2001; Shyeed et al., 2023; Rahman et al., 2025). We calculated the mean, median, and standard deviation of six physicochemical properties (Figure S2). Over 50% of alkaloids in AlkaPlorer satisfy RO5 criteria, underscoring their strong potential as orally bioavailable drug candidates. Comparative analysis of terpenoids and small‐molecule drugs across six physicochemical properties revealed distinct profiles for alkaloids. In molecular weight (MW), LogP, and topological polar surface area (TPSA), alkaloids demonstrated closer statistical alignment with approved small‐molecule drugs. Regarding hydrogen‐bond acceptors (HBA) and hydrogen‐bond donors (HBD), alkaloids exhibited significantly higher counts than terpenoids, likely due to nitrogen's dual capacity as both an efficient hydrogen bond donor and acceptor. This drug‐like profile, combined with their diverse bioactivities and natural origin, positions alkaloids as a privileged scaffold class for drug discovery.

Figure 5D summarizes the activity profiles of the top 10 classes identified from 17,205 bioactive alkaloids, with anticancer and anti‐infective effects predominating across these structural classes, although their relative prevalence varied considerably among different alkaloid categories. Notably, anti‐infective research records showed significantly higher proportions in Pyridine, Pyrrolidine, Piperazine, and Quinoline alkaloids, whereas anticancer research records dominated in Pyrimidine, Isoquinoline, and Indole alkaloids. We also analyzed the top 10 most abundant targets across three categories: single proteins, cell lines, and organisms. Acetylcholinesterase emerges as the most targeted enzyme with 925 alkaloid interactions. Cytochrome P450 isoforms (3A4/2D6/2C9/2C19/1A2) collectively account for 3,254 entries, indicating extensive alkaloid modulation of xenobiotic metabolism. SARS‐CoV‐2 replicase polyprotein 1ab ranks fifth with 681 hits, which may be attributed to a shift in research focus driven by the COVID‐19 pandemic. Nevertheless, this observation still highlights the potential of alkaloids in the development of viral protease inhibitors. The top 10 cell lines represent widely used *in vitro* models for evaluating anticancer agents (Table S2). Their prevalence spans major cancer types, including lung (A549), breast (MCF7/MDA‐MB‐231), colorectal (HCT‐116/HT‐29), leukemia (HL‐60/K562), and cervical (HeLa) malignancies (Table S3). Organism targets encompass major human pathogens (*S. aureus*, *P. falciparum*, *E. coli*) and model organisms critical to antimicrobial research (Table S4). The high‐frequency investigation of antimicrobial activities in tissue targets and anticancer studies in cell line models aligns with the elevated antibacterial/anticancer research frequencies observed in Figure 2B.

### Webserver

**Figure 6:**
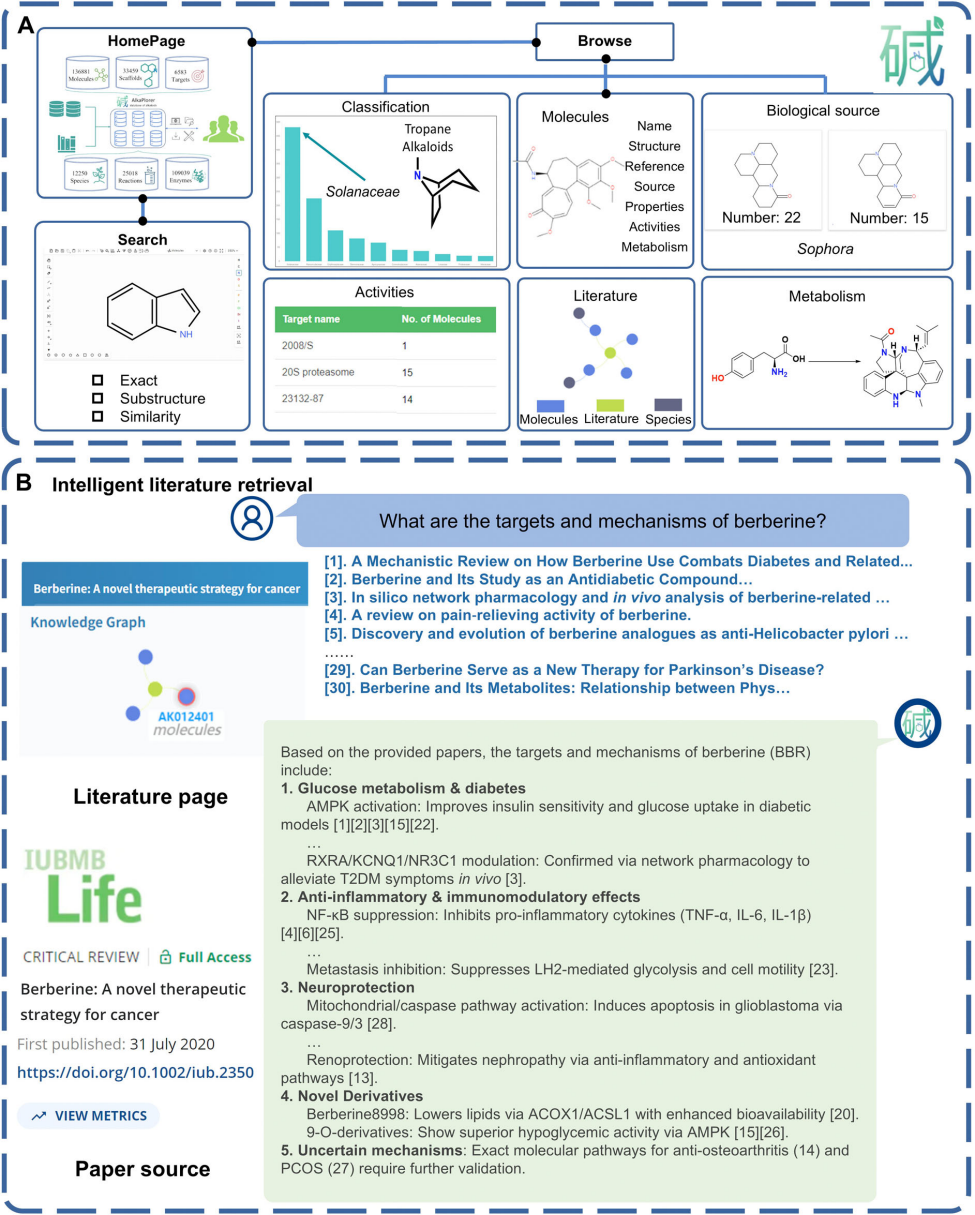
Core functional interfaces of the AlkaPlorer platform **(A)** Homepage, Browse windows, and Search window. **(B)** Illustration of the artificial intelligence (AI)‐powered intelligent literature retrieval.

A web‐based platform, AlkaPlorer (https://alkaplorer.qmclab.com/), was established to facilitate the exploration of alkaloids through integrated browsing, searching, and analytical functionalities, as shown in Figure 6. The homepage provides a user‐friendly interface for browsing. For structure‐centric exploration, the Structure Search module provides a chemical editor for direct structure drawing or input via SMILES/InChI string pasting, supporting exact, substructure, and similarity‐based queries. The Advanced Search interface focuses on physicochemical property‐driven queries, allowing users to filter alkaloids by parameters, such as LogP, molecular weight, polar surface area, and hydrogen bond donor/acceptor counts. These criteria support drug discovery workflows, enabling rapid prioritization of compounds with desirable pharmacokinetic profiles.

The platform offers users multiple browsing modules, enabling systematic exploration of alkaloids from various perspectives. Users interested in the distribution of a specific alkaloid class can browse compounds through the classification and scaffold pages, and then connect these results to the biological source and activity distribution. For a more specific case, as shown in Figures 6A, S3, users who are interested in tropane alkaloids can browse the page of tropane alkaloids to not only identify specific compounds but also explore the distribution of biological source and target of these alkaloids that are predominantly sourced from the Solanaceae family, with additional occurrences reported in Ranunculaceae, Erythroxylaceae, and Apocynaceae, consistent with findings from comprehensive reviews (Afewerki et al., 2019). In the meantime, users can also browse by biological source or target. Selecting the genus *Sophora* reveals its alkaloid profiles dominated by quinolizidine and quinolizidine‐like scaffolds (e.g., quinolizidine rings), aligning with the characteristic biosynthetic pathways of this taxon (Cai et al., 2020).

AlkaPlorer provides multiple search options, allowing users to locate a target alkaloid through different entry points. In addition to the standard structure search and filtering functions described above, we implemented an intelligent literature retrieval module to support more reliable user interactions (Figure 6B). Upon receiving a question, the retrieval tool first searches the AlkaPlorer database to identify relevant scientific references. The abstracts of these retrieved documents are then passed to a Large Language Model (LLM). The LLM synthesizes its response strictly based on the content of these provided abstracts. This process ensures that every statement in the final answer can be directly supported by existing literature, thereby significantly enhancing the trustworthiness of the LLM's output and reducing the incidence of hallucinations. Additionally, the platform provides direct access to relevant scientific literature, integrating a knowledge graph that visually connects these publications to associated molecules, facilitating deeper insight into structural and functional relationships. Through cross‐linked browsing sections, data interoperability is strengthened, offering users a more intuitive way to explore and understand data relationships. To illustrate the workflow for retrieving a specific alkaloid in AlkaPlorer, we use berberine as an example. Users can locate berberine via structure search or by applying custom filters in the Advanced Search page to retrieve related compounds beyond berberine itself. Alternatively, the intelligent literature retrieval module can be used to summarize literature information relevant to berberine (Figures 6B, S4). The retrieved references can be opened by clicking into the corresponding detail pages, where the knowledge graph provides another route to navigate back to the berberine molecule. On the berberine detail page, users can explore over 100 species that produce berberine, originating from families such as Berberidaceae, Papaveraceae, and Menispermaceae, hundreds of bioactivity records detailing its effects on various biological targets, including its effects on enzyme inhibition (e.g., acetylcholinesterase, cytochrome P450), immune modulation, anti‐inflammatory and antimicrobial activities, as well as antiviral effects (e.g., against SARS‐CoV‐2 and Hepatitis C virus), and reaction data providing synthesis information for various berberine derivatives. All this information is supported by external links or literature, offering users a one‐stop, traceable resource for alkaloid exploration.

## DISCUSSION

**Figure 7:**
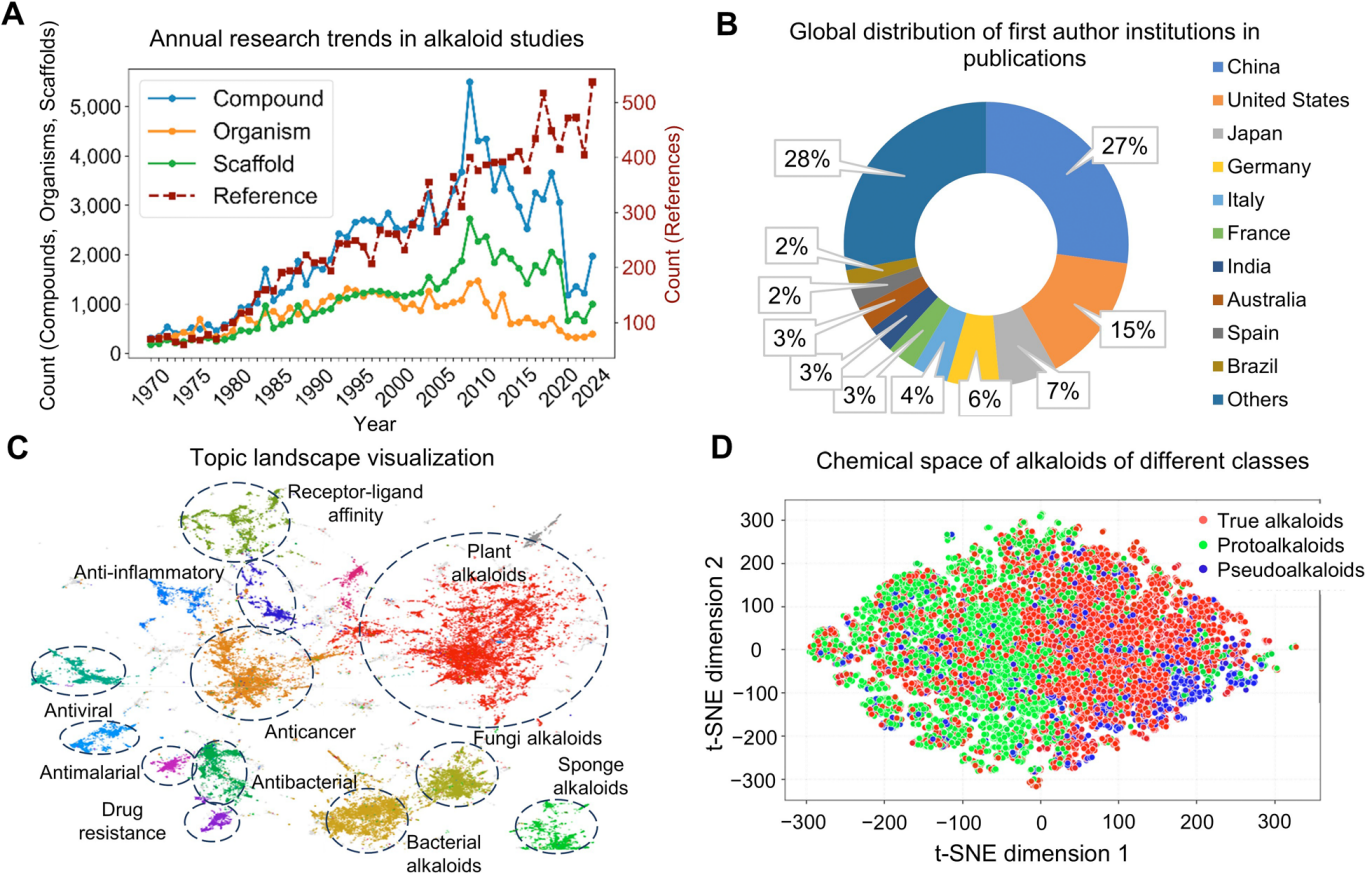
Scientometric analysis of alkaloid research trends and chemical space visualization **(A)** Annual trends in alkaloid studies (1970–2024). The number of instances for compounds, organisms, scaffolds, and references related to alkaloids was extracted from the literature and plotted as a function of year. **(B)** Distribution of country of origin for publications on the topic of alkaloids. The institution of the first author was used to generate this plot. **(C)** Visualization of the research topics derived from the titles and abstracts of the curated literature in AlkaPlorer, generated using the BERTopic framework. **(D)** Chemical space of three alkaloid classes based on 2048‐bit Morgan fingerprints (radius 2) calculated via RDKit, visualized using t‐SNE dimensionality reduction.

Alkaloids are an important family in NPs and exhibit huge potencies in drug discovery. More and more alkaloids have been isolated and determined from the natural world in the last 200 years. To optimize alkaloid literature management and analyze research trends, we designed the alkaloid literature management module, which classifies database literature into two major themes: structure discovery and bioactivity. The history of alkaloid discovery from 1975 to 2024 can be interpreted as a tension between technological innovation and biological source depletion. The fluctuating upward trend in publications on alkaloid discovery underscores sustained interest in alkaloids, likely driven by their profound pharmacological significance (Figure 7A). The initial rise of alkaloid discovery to the peak (around 2009) can be fueled by technology (e.g., NMR, LC‐MS), enabling the rapid characterization of compounds from easily accessible biological material. However, the subsequent plateau and decline expose a critical underlying constraint: the steady decline in novel biological sources since the early 1990s. As the pipeline of new organisms dried up, the field became increasingly dependent on re‐mining known organisms. While this sustained discovery for a time (the plateau), it ultimately led to a period of decline as the low‐hanging fruit in these sources was exhausted (the COVID‐19 pandemic is likely to be an important exacerbating factor as well). The nascent recovery observed by 2024 is intriguing and may signal the initial impact of next‐generation strategies, such as genome mining and metabolomics, which are now beginning to unlock the “dark matter” of previously undetectable alkaloids from well‐studied sources. We further analyzed the affiliations of 22,734 publications, which were easily obtained via DOI. The global landscape of alkaloid research demonstrates a distinct trend toward consolidation, led by China (Figure 7B). Notably, collaborative efforts across borders play a significant role in this field, with 32.74% of the published articles co‐authored by researchers from different countries. This trend underscores both the advantages of resource concentration and scale and potentially indicates a bias in research paradigms and geographical origins. For the future development of the field, it is imperative to strike a better balance between the efficiency gained from this centralization and the breadth of global biodiversity exploration, alongside equity in collaboration. Using the embeddings generated by Qwen3‐embedding, we performed clustering analysis, which identified 16 distinct thematic clusters. Each cluster was manually reviewed and labeled based on its constituent articles, and the overall distribution of these topics is visualized in Figure 7C, and the characteristic keywords for each topic are listed in Figure S5. In addition to the topics we have previously discussed, such as plant alkaloids and anticancer research, several other topics explore discussions on drug resistance, including antibiotic resistance and its mechanisms, such as drug efflux mediated by transporters like P‐glycoprotein (P‐gp). We employed Morgan fingerprints by RDKit to represent molecular structures, followed by t‐distributed stochastic neighbor embedding (t‐SNE) for dimensionality reduction to two dimensions, enabling visualization of the alkaloid chemical space. As shown in Figure 7D, the chemical spaces of true alkaloids, protoalkaloids, and pseudoalkaloids are fundamentally distinct, owing to their differential nitrogen incorporation and distinct biosynthetic precursors. Furthermore, bioactive alkaloids are not clustered in a specific region but are scattered throughout the entire chemical space, indicating that active compounds may originate from diverse alkaloid scaffolds rather than being uniformly distributed (Figure S6). This non‐uniform distribution, yet not entirely random, also suggests that bioactive compounds exhibit certain structural preferences. Regarding biological origins, when classified into Viridiplantae, Metazoa, Fungi, and Bacteria, all four categories exhibit structural diversity and broad distribution. However, distinct patterns are observed among them, localized regional disparities exist among alkaloids from these different biological sources.

Compared to other natural products database (Chen et al., 2025), AlkaPlorer addresses key limitations of existing natural product databases by systematically collecting alkaloid data and organizing them using a unified and hierarchical classification framework, supported by extensive manual curation. Beyond standardized structural and biological source annotations, the database establishes comprehensive links to literature‐derived bioactivity data and reaction information, enabling integrated analyses of alkaloid function and metabolism. In addition, AlkaPlorer provides real‐time statistical analysis and multidimensional browsing capabilities, allowing users to dynamically explore alkaloid distributions across chemical classes, biological sources, and activity profiles (Table S5).

While the database currently contains records from 2019 to 2024 that have been meticulously manually verified, earlier records (pre‐2019) are incorporated from public databases as a foundational starting point. Our ongoing efforts are focused on progressively applying the same rigorous curation standards to these historical entries. Additionally, the current classification approach is based on the commonly reported rules in the literature, as the biosynthetic pathways for each alkaloid molecule are not yet fully comprehensive. In the future, we aim to construct a complete metabolic network for alkaloids to enable more precise precursor classification. We have a clear roadmap to address these aspects by integrating advanced LLM and cheminformatics tools, which will significantly accelerate both the validation and enrichment of data, continuously enhancing the resource's depth and accuracy. Concurrently, the database will be regularly updated, with ongoing development of specialized tools to expand its utility in chemistry, synthetic biology, and pharmaceutical sciences.

In summary, we established a user‐friendly database, AlkaPlorer, which includes the chemical structures, physicochemical properties, biogenesis structure tree, biological sources, activities, and metabolism information of alkaloids by integrating and annotating the accumulated data over the past few decades. The systematic categorization and analysis lay the foundation for exploring the chemical space of alkaloids from an evolutionary biogenesis perspective, offering new insights into the diversification and ecological roles of these NPs. Additionally, the user‐friendly webserver allows for more refined and personalized analyses, facilitating progress across the entire field. We believe that AlkaPlorer will serve as an essential platform for accelerating alkaloid‐centric structure discovery and drug development.

## MATERIALS AND METHODS

### Data preparation

To compile comprehensive chemical and biological data on alkaloids, we systematically aggregated information from multiple established sources, including DNP, COCONUT (Chandrasekhar et al., 2025), NPASS (Zhao et al., 2023), Lotus (Rutz et al., 2022), and The Natural Products Atlas (van Santen et al., 2019). The initial dataset comprised approximately 0.6 million nitrogen‐containing compounds. To ensure data integrity and research relevance, we implemented a rigorous three‐step curation protocol: (1) To prioritize high‐confidence records and minimize potentially spurious entries, we retained only compounds supported by either identified biological sources or supporting literature; (2) to remove unrecognizable or erroneous structures, molecules with incomplete structural information (missing bond data or invalid SMILES strings) were excluded to ensure structural accuracy; and (3) to ensure a non‐redundant dataset, duplicate molecules were collapsed using InChIKey as a unique molecular identifier, retaining only one representative entry per InChIKey. The numbers of records retained after each step are summarized in Table S6. In the meantime, to expand coverage of newly reported alkaloids, we searched PubMed, Web of Science and Scopus using “alkaloids” as the keyword from 2019 to 2024 and then used DeepSeek‐R1 to select 2,367 papers reporting new alkaloids. In order to assess the classification performance of DeepSeek‐R1 in identifying papers reporting new alkaloids, we manually selected 50 positive samples (papers reporting new alkaloids) and 50 negative samples (papers that did not report new alkaloids). As summarized in Tables S7 and S8, DeepSeek‐R1 successfully identified the majority of papers reporting new alkaloids. We manually extracted all newly discovered alkaloids curated by experts familiar with alkaloids from these papers, including structural information, biological source, and classification within each paper.

Following structural standardization, all entries were processed using RDKit (v2023.09.6) by computing InChI, InChIKey, and Murcko scaffolds, and more than 200 property descriptors (e.g., molecular weight, TPSA) for property‐based querying and downstream analyses. Morgan fingerprints (radius = 2, nBits = 2,048) were also generated to enable similarity search and clustering. The database employs a biogenesis‐structure taxonomy (Table S9) for hierarchical alkaloid classification. Level 1 differentiates true alkaloids (containing heterocyclic nitrogen atoms biosynthesized from amino acids, e.g., morphine), protoalkaloids (containing non‐heterocyclic nitrogen compounds biosynthesized from amino acids like colchicine), and pseudoalkaloids (containing non‐amino acid precursor‐derived compounds such as purine alkaloids) (Figure S7). Level 2 maps biosynthetic precursors (e.g., tryptophan‐derived alkaloids), while Level 3 defines core structural scaffolds (e.g., indole or isoquinoline frameworks). Representative alkaloids are further subclassified into Level 4 and Level 5 to capture fine‐grained structural categories. This multi‐tiered system enables systematic exploration from biosynthetic origins to structural refinements, facilitating drug discovery workflows and comprehensive chemical space mapping of alkaloid diversity.

Biological sources were normalized from heterogeneous descriptions by entity extraction from free‐text records (e.g., DNP ‘Prod. by … *Phomopsis longicolla* HL‐2232’) and DeepSeek‐R1 ‐ assisted inference for manually curated literature, followed by expert validation; validated taxa were mapped to the NCBI Taxonomy Database (Schoch et al., 2020) to obtain standardized hierarchical lineages (domain, kingdom, phylum, class, order, family, genus, species). Through InChIKey‐based matching, ChEMBL (Davies et al., 2015; Zdrazil et al., 2024) provided validated bioactivity parameters (target names, organism, activity type/value/units and references) for ligand‐target interactions, whereas MetaCyc (Caspi et al., 2020), KEGG (Kanehisa and Goto, 2000) and UniProt (Coudert et al., 2023) collectively curated metabolic pathway data, including enzymatic reaction annotations and catalytic enzyme associations.

### Web server implementation

AlkaPlorer was architected as a relational database using PostgreSQL 11.2 for backend management, enabling scalable storage and efficient querying of alkaloid‐related chemical and biological data. The system supports advanced cheminformatics searches, including exact match, substructure, and similarity‐based queries, through integration with the RDKit cheminformatics toolkit.

The web interface, deployed on a Linux server (CentOS Linux 8 Core), was built using Flask 3.0.3 with Nginx as the reverse proxy. Frontend components were developed with HTML5, CSS3, and JavaScript ES6, while the chemical structure visualization was implemented via ChemDoodle Web Components 10.0.0, ensuring SMILES compatibility and responsive 2D and 3D rendering, and molecular editing functionalities were supported by Ketcher 3.2.0. Under a 50‐concurrent‐client load, representative queries showed a mean response time of 3.95 s, suggesting that the current deployment is sufficiently responsive for routine use by research users.

Seventy seven thousand nine hundred and sixty three curated abstracts documenting novel alkaloid discoveries and bioactivity profiles from AlkaPlorer form the knowledge base, and the Qwen text‐embedding‐v4 model from Alibaba Cloud was used for knowledge embedding (Table S10). The system employs DeepSeek‐R1‐0528 for output generation, ensuring optimal text synthesis capabilities.

## CONFLICTS OF INTEREST

The authors declare no conflicts of interest.

## AUTHOR CONTRIBUTIONS

R.W. designed and supervised the whole research. L.J., Z.T., X.H., and K.X. jointly conceived the research framework, designed the methodology, and established the workflow. L.J. collected, cleaned, and annotated the data. L.J. and Z.T. statistically analyzed the results. L.M. and L.J. collected and analyzed the trend of alkaloid research. L.J. and Z.T. wrote and revised the manuscript. All authors read and approved the manuscript.

## Supporting information

Additional Supporting Information may be found online in the supporting information tab for this article: http://onlinelibrary.wiley.com/doi/10.1111/jipb.70173/suppinfo

**Figure S1**. Number of alkaloids from the top 10 families in Viridiplantae, Fungi, Bacteria, and Metazoa
**Figure S2**. Statistical distribution of descriptors for alkaloids in AlkaPlorer
**Figure S3**. Step‐by‐step guide for exploring tropane alkaloids and the alkaloid profiles of the genus *Sophora* in AlkaPlorer
**Figure S4**. Step‐by‐step guide for retrieving a specific alkaloid, using berberine as an example, through three different search methods in AlkaPlorer
**Figure S5.** Top keyword distribution across themes
**Figure S6**. Comparative Chemical Space Analysis of the Alkaloid Library
**Figure S7**. Examples of true alkaloids, protoalkaloids, and pseudoalkaloids
**Table S1.** Bioactivity data in AlkaPlorer: categories, activity records, and alkaloid counts
**Table S2.** Top 10 single proteins by bioactivity alkaloids abundance
**Table S3.** Top 10 cell lines by bioactivity alkaloids abundance
**Table S4.** Top 10 organisms by bioactivity alkaloids abundance
**Table S5.** Systematic functional comparison between AlkaPlorer and major natural product databases that include alkaloids (COCONUT, NPASS, LOTUS, GNDC, and DNP)
**Table S6.** Summary of record retention after each step of the three‐step curation pipeline
**Table S7.** Evaluation of DeepSeek‐R1 for identifying papers reporting new alkaloids
**Table S8.** Classification performance metrics of DeepSeek‐R1 in detecting newly reported alkaloids
**Table S9.** Key structures in the biosynthesis structure tree
**Table S10.** Parameters of the embedding model

## Data Availability

All structural data can be accessed through the Data page of AlkaPlorer (https://alkaplorer.qmclab.com/data).
