# Supplementary material for: AlkaPlorer: A database‐driven explorer for natural alkaloids and derivatives
Source: J Integr Plant Biol. 2026 Feb 5;68(5):1384–98. doi: 10.1111/jipb.70173 (PMC13140036; doi:10.1111/jipb.70173)
Supplement: Supplementary file 1 — Figure S1. Number of alkaloids from the top 10 families in Viridiplantae, Fungi, Bacteria, and Metazoa Figure S2. Statistical distribution of descriptors for alkaloids in AlkaPlorer Figure S3. Step‐by‐step guide for exploring tropane alkaloids and the alkaloid profiles of the genus Sophora in AlkaPlorer Figure S4. Step‐by‐step guide for retrieving a specific alkaloid, using berberine as an example, through three different search methods in AlkaPlorer Figure S5. Top keyword distribution across themes Figure S6. Comparative Chemical Space Analysis of the Alkaloid Library Figure S7. Examples of true alkaloids, protoalkaloids, and pseudoalkaloids Table S1. Bioactivity data in AlkaPlorer: categories, activity records, and alkaloid counts Table S2. Top 10 single proteins by bioactivity alkaloids abundance Table S3. Top 10 cell lines by bioactivity alkaloids abundance Table S4. Top 10 organisms by bioactivity alkaloids abundance Table S5. Systematic functional comparison between AlkaPlorer and major natural product databases that include alkaloids (COCONUT, NPASS, LOTUS, GNDC, and DNP) Table S6. Summary of record retention after each step of the three‐step curation pipeline Table S7. Evaluation of DeepSeek‐R1 for identifying papers reporting new alkaloids Table S8. Classification performance metrics of DeepSeek‐R1 in detecting newly reported alkaloids Table S9. Key structures in the biosynthesis structure tree Table S10. Parameters of the embedding model [file JIPB-68-1384-s001.docx]

**AlkaPlorer: A Database-driven Explorer for Natural Alkaloids and Derivatives**

Jiahao Li^1,†^, Tao Zeng^2,†^, Hongquan Xu^1^, Xu Kang^1^, Minghui Liang^3^, Ruibo Wu^1,^*

^1^ School of Pharmaceutical Sciences, Sun Yat-sen University, Guangzhou 510006, China

^2^ School of Pharmaceutical Sciences, Hainan University, Haikou 570228, China

^3^ School of Pharmaceutical Sciences, Guangzhou Medical University, Guangzhou 511436, China

†These authors contributed equally to this work.

*Correspondence: Ruibo Wu ([wurb3@mail.sysu.edu.cn](mailto:wurb3@mail.sysu.edu.cn))

**Table S1**. **Bioactivity data in AlkaPlorer: categories, activity records, and alkaloid counts.**

| Target type | Counts | Alkaloids |
| --- | --- | --- |
| Organism | 413,652 | 8,236 |
| Single Protein | 224,480 | 6,540 |
| Cell Line | 177,498 | 9,182 |
| Others | 144,049 | 8,508 |

**Table S2**. **Top 10 single protein by bioactivity alkaloids abundance**

| Target name | Alkaloids |
| --- | --- |
| Acetylcholinesterase | 925 |
| Tyrosyl-DNA phosphodiesterase 1 | 863 |
| Cytochrome P450 3A4 | 727 |
| Histone deacetylase 6 | 704 |
| Replicase polyprotein 1ab(SARS-CoV-2) | 681 |
| Cytochrome P450 2D6 | 632 |
| Cytochrome P450 2C9 | 606 |
| Cytochrome P450 2C19 | 592 |
| Monoamine oxidase A | 591 |
| Cytochrome P450 1A2 | 589 |

**Table S3**. **Top 10 cell lines by bioactivity alkaloids abundance**

| Cell-Line | Alkaloids |
| --- | --- |
| A549 | 2,586 |
| MCF7 | 2,098 |
| HCT-116 | 1,432 |
| HL-60 | 1,326 |
| HT-29 | 1,224 |
| HepG2 | 1,205 |
| MDA-MB-231 | 1,188 |
| K562 | 1,154 |
| NCI-H460 | 1,146 |
| HeLa | 1,024 |

**Table S4**. **Top 10 organism by bioactivity alkaloids abundance**

| Organism | Alkaloids |
| --- | --- |
| Staphylococcus aureus | 2,169 |
| Plasmodium falciparum | 2,059 |
| Escherichia coli | 1,830 |
| Candida albicans | 1,396 |
| SARS-CoV-2 | 1,064 |
| Pseudomonas aeruginosa | 1,038 |
| Bacillus subtilis | 948 |
| Mus musculus | 900 |
| Rattus norvegicus | 738 |
| Mycobacterium tuberculosis | 682 |

**Table S5**. **Systematic functional comparison between AlkaPlorer and major natural product databases that include alkaloids (COCONUT, NPASS, LOTUS, GNDC and DNP).**

| Function/Database | AlkaPlorer | COCONUT | NPASS | LOTUS | DNP | GNDC |
| --- | --- | --- | --- | --- | --- | --- |
| Numbers of alkaloids | 136,881 | 169,393 ^a^ | Not directly accessible ^b^ | Not directly accessible ^b^ | Access restricted ^c^ | 492,014 |
| Presence of literature evidence | Fully supported | Partially supported | Fully supported | Fully supported | Access restricted | N/A |
| Real-time data statistical analysis | Available | N/A | N/A | N/A | N/A | N/A |
| Systematic classification of alkaloids | Available | N/A | N/A | N/A | N/A | N/A |
| Reactions information | Available | N/A | N/A | N/A | N/A | N/A |
| Activities Information (Literature-based) | Available | N/A | Available | N/A | N/A | N/A |
| Activities Information (Predicted) | N/A | N/A | Available | N/A | N/A | Available |
| Intelligent literature retrieval function | Available | N/A | Available | N/A | N/A | N/A |
| Latest update of the database | 2025-NOV | 2024-NOV | 2025-JUN | 2021-MAY | 2025 | 2025-APR |
| All database features and data were accessed by webserver on December 20, 2025. | | | | | | |

a. COCONUT originally contained 186,359 alkaloids. After removing entries flagged as “REVOKED” due to insufficient evidence, 169,393 alkaloids remained.

b. “Not directly accessible” indicates that LOTUS and NPASS do not provide a dedicated interface or page that allows direct access to all alkaloid entries within the database.

c. DNP is a commercial database and requires a paid subscription for access; it is not open source.

**Table S6**. **Summary of record retention after each step of the three-step curation pipeline**

| Steps | Record retention |
| --- | --- |
| First collection | 599,355 |
| Evidence filtering | 180,844 |
| Structure validation | 142,425 |
| InChIKey-based deduplication | 129,501 |
| After manual extraction | 136,881 |

As **Table S6** and **Table S7** shows, to assess the classification performance of DeepSeek-R1, we manually curated a benchmark set consisting of 50 positive samples (papers explicitly reporting newly discovered alkaloids) and 50 negative samples. DeepSeek-R1 correctly identified 49 out of 50 positive papers and 46 out of 50 negative papers. The resulting performance metrics were: 95.0% accuracy, 92.5% precision, 98.0% recall, and 95.1% F1-score. These results indicate that DeepSeek-R1 provides high reliability for initial large-scale screening, although all final decisions were still confirmed by manual expert curation.

**Table S7**. **Evaluation of DeepSeek-R1 for identifying papers reporting new alkaloids.**

|  | Actual positive (*n* = 50) | Actual negative (*n* = 50) |
| --- | --- | --- |
| Predicted positive | 49 | 46 |
| Predicted negative | 1 | 4 |

**Table S8**. **Classification performance metrics of DeepSeek-R1 in detecting newly reported alkaloids.**

| Metric | Value |
| --- | --- |
| Accuracy | 95.0% |
| Precision | 92.5% |
| Recall | 98.0% |
| F1-score | 95.1% |

**Table S9**. **Key Structures in Biosynthesis structure Tree**

| Class name | Key Structure |
| --- | --- |
| True alkaloids | Biosynthesized from amino acid and containing heterocyclic nitrogen atoms. |
| Protoalkaloids | containing non-heterocyclic nitrogen compounds biosynthesized from amino acids. |
| Pseudoalkaloids | containing non-amino acid precursor-derived compounds. |
| L-tyrosine or L-phenylalanine-derived alkaloids |  |
| L-ornithine-derived alkaloids |  |
| L-lysine-derived alkaloids |  |
| L-histidine-derived alkaloids |  |
| L-tryptophan-derived alkaloids |  |
| Aspartate-derived alkaloids |  |
| Anthranilic acid-derived alkaloids |  |
| Indole alkaloids |  |
| Quinoline alkaloids |  |
| Pyrroloindole alkaloids |  |
| Pyrimidine-containing alkaloids |  |
| Isoquinoline alkaloids |  |
| Tropane alkaloids |  |
| Pyrrolizidine alkaloids |  |
| Pyrrolidine alkaloids |  |
| Quinolizidine alkaloids |  |
| Piperidine alkaloids |  |
| Pyridine alkaloids |  |
| Quinazoline alkaloids |  |
| Acridone alkaloids |  |
| Amino acid alkaloids |  |
| Piperazine alkaloids |  |
| Imidazole alkaloids |  |
| Oxazole alkaloids |  |
| Thiazole alkaloids |  |
| Nitriles alkaloids |  |
| Pteridine alkaloids |  |
| Diazene-containing alkaloids |  |
| Nitro group-containing alkaloids |  |
| Phenazine alkaloids |  |
| Arginine-derived alkaloids |  |
| Guanidine alkaloids |  |
| Tetrahydroisoquinoline alkaloids |  |
| Phenoxazine alkaloids |  |
| Purine alkaloids |  |
| Iboga type |  |
| Monoterpene indole alkaloids |  |
| Corynanthe |  |
| Aspidosprma |  |
| Benzylisoquinoline alkaloids |  |
| Bisbenzylisoquinoline alkaloids |  |
| Aporphine isoquinoline alkaloids |  |
| Naphthylisoquinoline alkaloids |  |
| Ergot alkaloids |  |
| Spirobenzylisoquinoline alkaloids |  |
| Stemona alkaloids |  |
| Indolizidine alkaloids |  |

**Table S10. Parameters of the embedding model.**

| Model | MTEB | MTEB (Retrieval task) | CMTEB | CMTEB (Retrieval task) |
| --- | --- | --- | --- | --- |
| Qwen Text-embedding-v4 (2048 dimensions) | 71.58 | 61.97 | 71.99 | 75.01 |
| Qwen3-Embedding-0.6B | 64.33 | 64.64 | 66.33 | 71.03 |


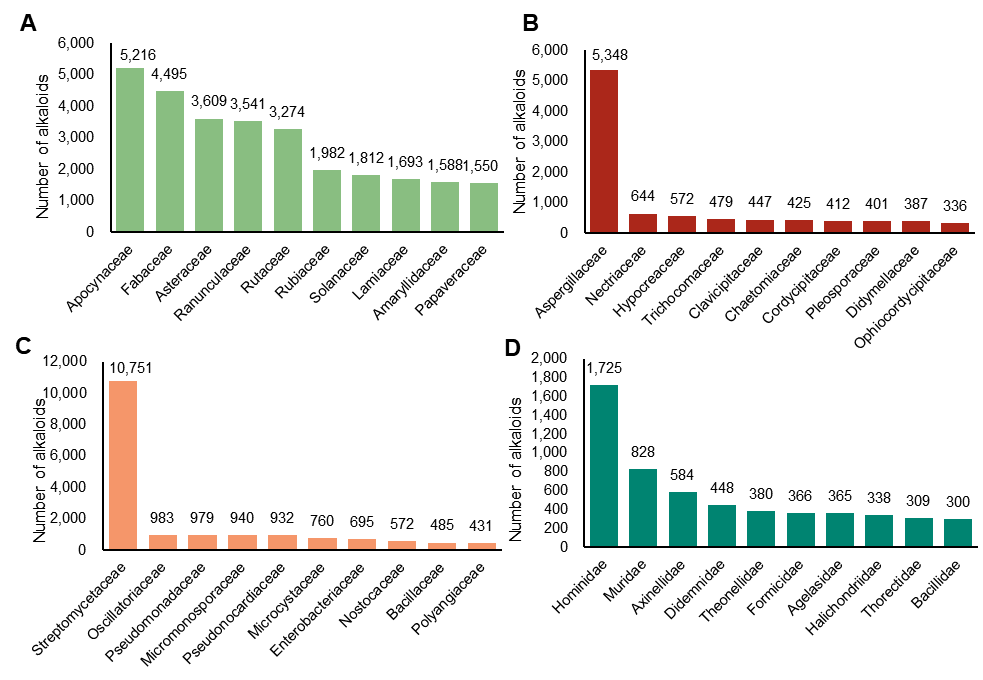


**Figure S1**. **Number of alkaloids from the top 10 families in (A) Viridiplantae, (B) Fungi, (C) Bacteria, and (D) Metazoa.**


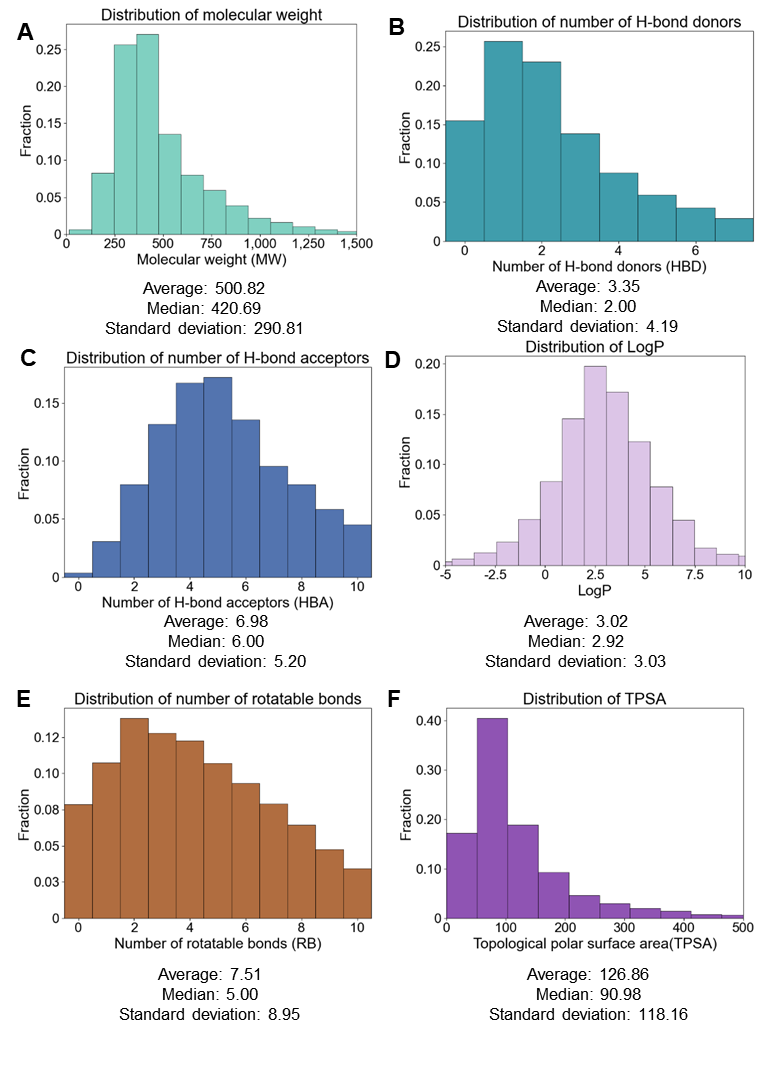


**Figure S2**. **Statistical distribution of descriptors for alkaloids in AlkaPlorer.**

**(A)** Molecular weight, **(B)** Number of H-bond donors, **(C)** Number of H-bond acceptors, **(D)** LogP, **(E)** Number of rotatable bonds, **(F)** Topological polar surface area (TPSA)


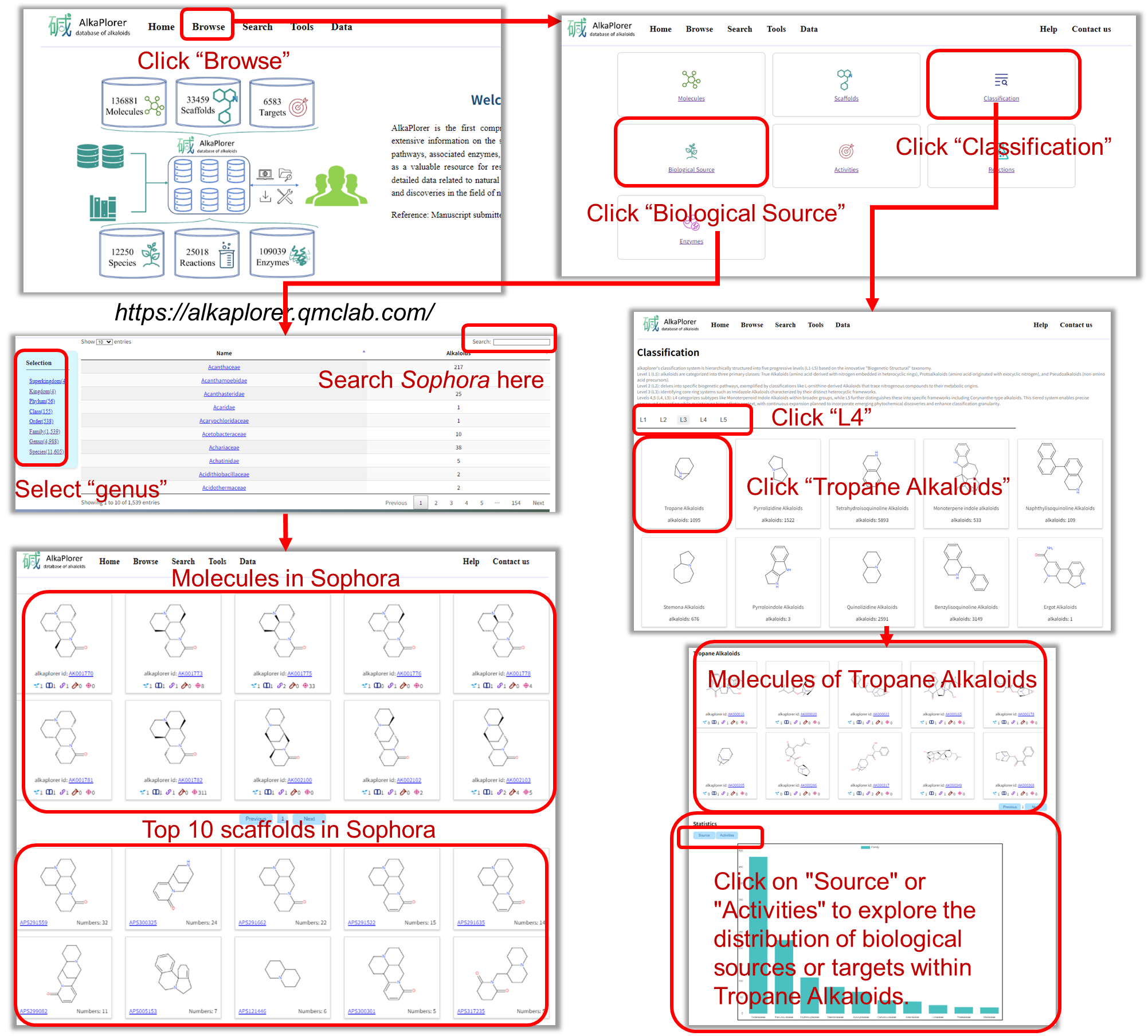


**Figure S3**. **Step-by-step guide for exploring tropane alkaloids and the alkaloid profiles of the genus *Sophora* in AlkaPlorer.**


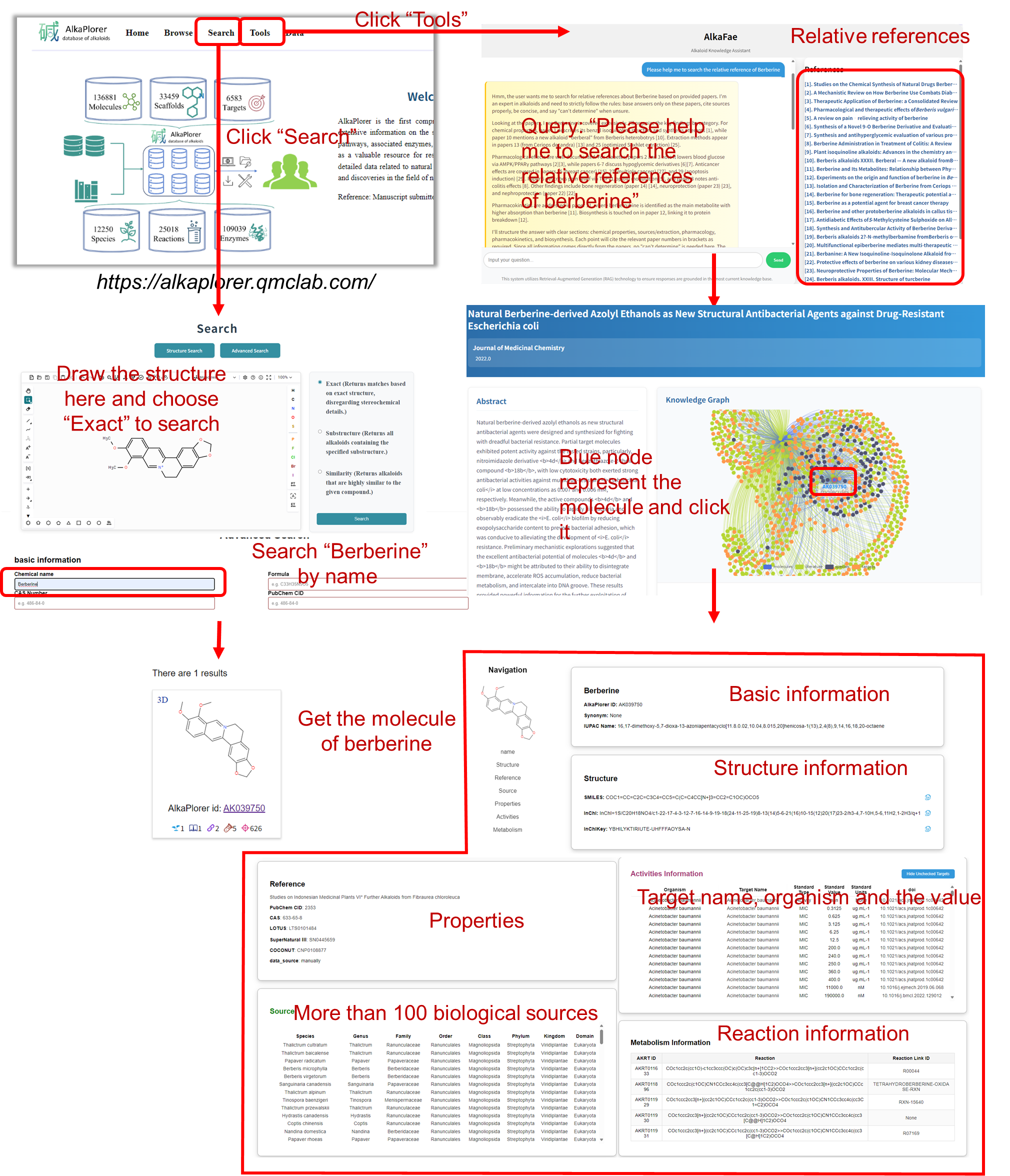


**Figure S4**. **Step-by-step guide for retrieving a specific alkaloid, using berberine as an example, through three different search methods in AlkaPlorer.**


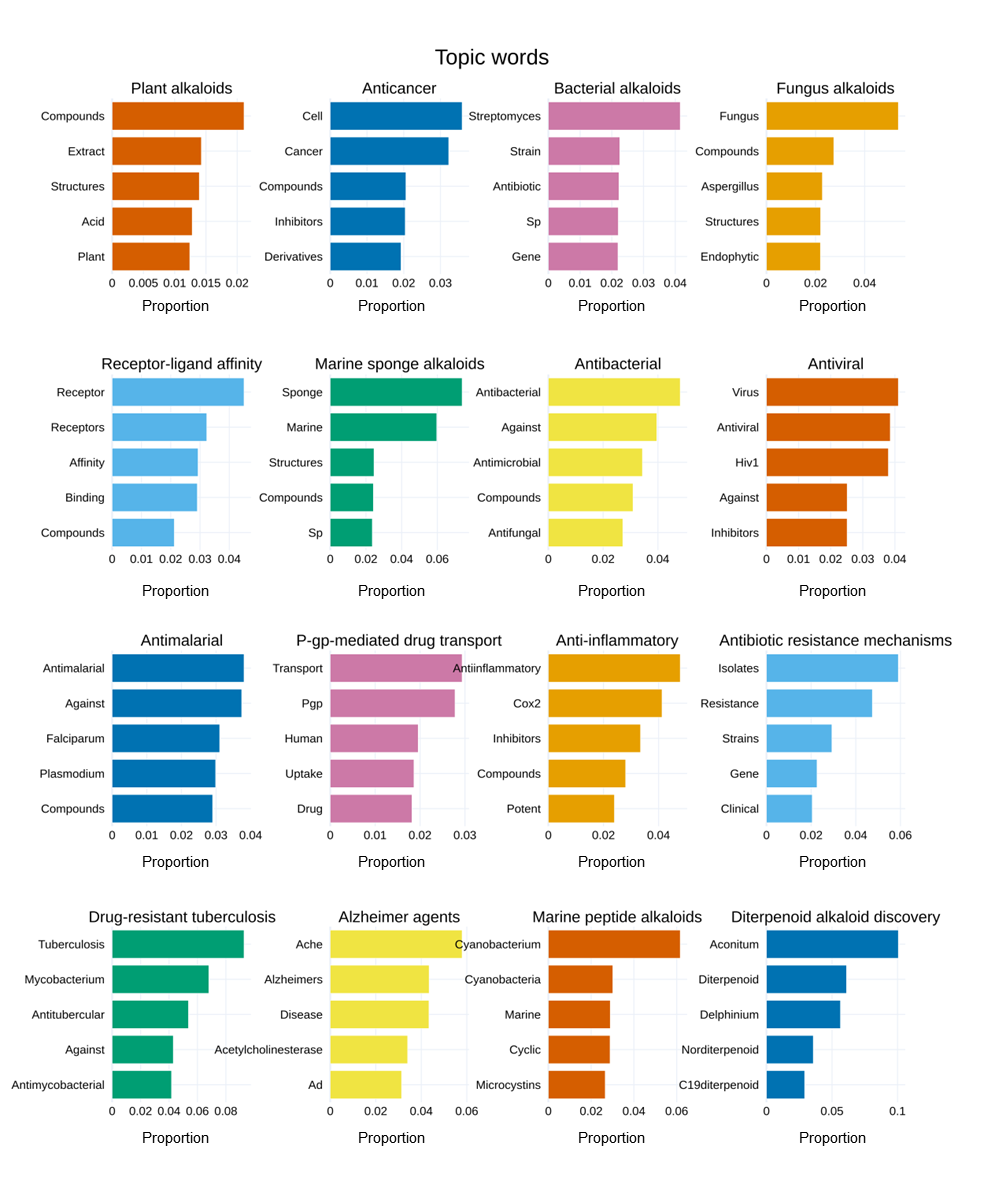


**Figure S5. ​Top keyword distribution across themes.**


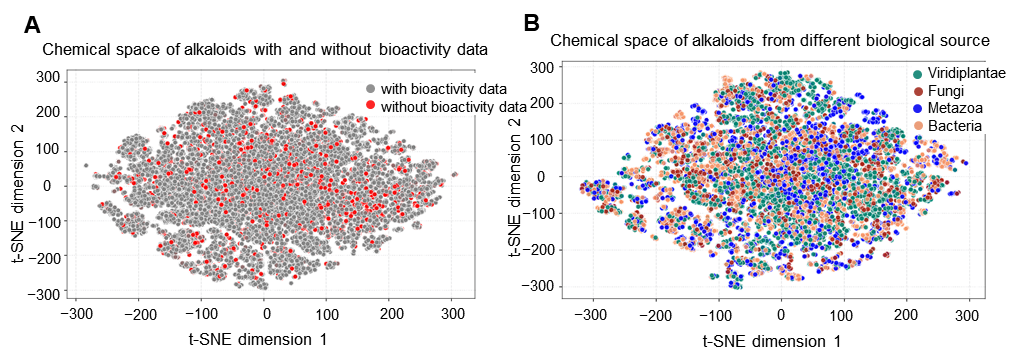


**Figure S6**. **Comparative chemical space analysis of the alkaloid library.**

**(A)** Chemical space of alkaloids with or without bioactivity data. **(B)** Chemical space of alkaloids from different biological source.


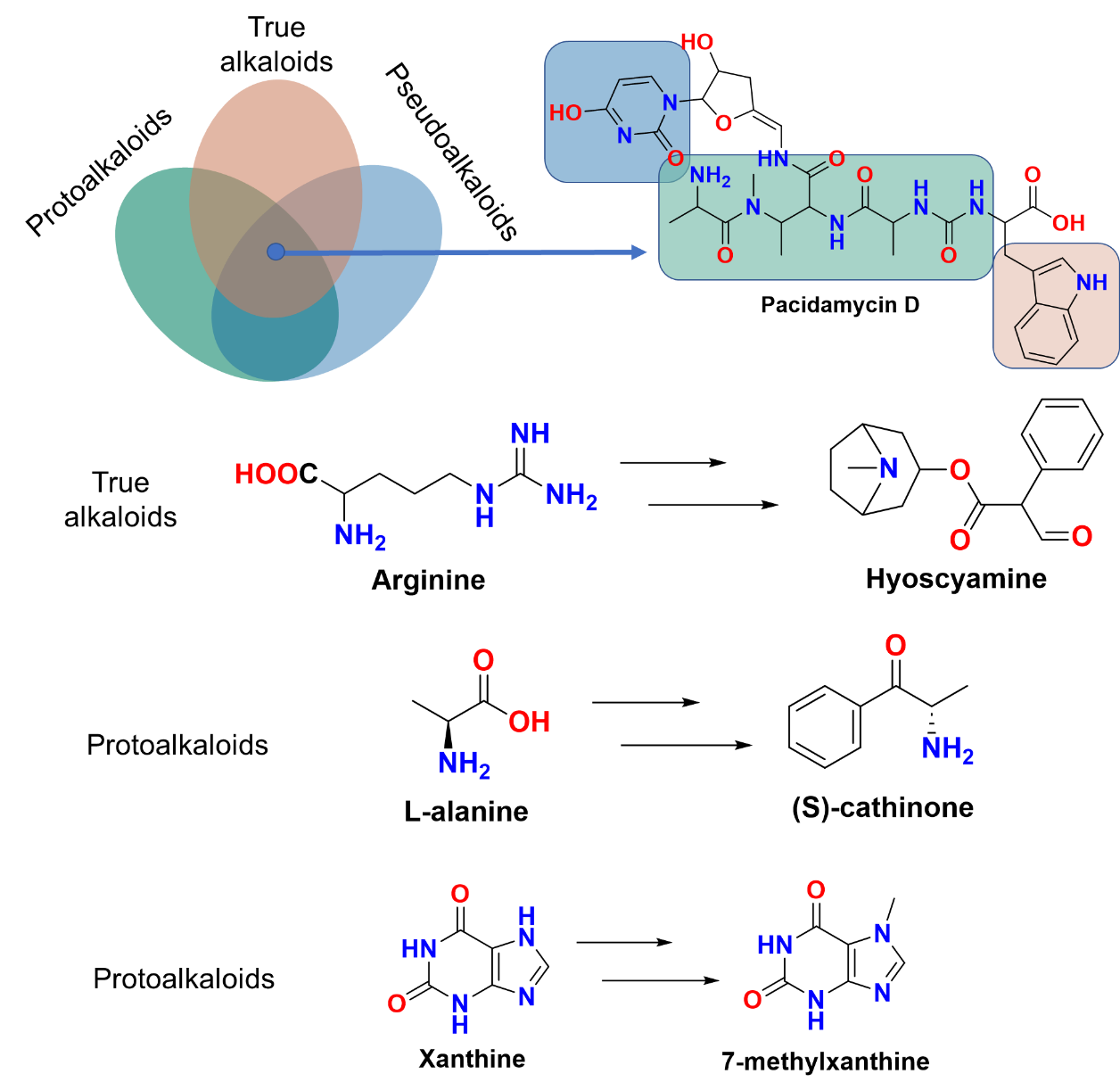


**Figure S7**. **Examples of true alkaloids, protoalkaloids and pseudoalkaloids.**
